# The impact of replacing milk with plant-based alternatives on iodine intake: a dietary modelling study

**DOI:** 10.1007/s00394-023-03286-7

**Published:** 2024-01-11

**Authors:** Katie Nicol, Anne P. Nugent, Jayne V. Woodside, Kathryn H. Hart, Sarah C. Bath

**Affiliations:** 1 Department of Nutrition, Food and Exercise Sciences, Faculty of Health and Medical Sciences, University of Surrey, Guildford, GU2 7XH UK; 2 Institute for Global Food Security, School of Biological Sciences, Queen’s University Belfast, Northern Ireland, UK; 3 Centre for Public Health, Queen’s University Belfast, Belfast, BT12 6BJ UK

**Keywords:** Iodine, Milk, Plant-based milk, Milk alternatives, Fortification

## Abstract

**Purpose:**

Cow’s milk is the primary source of iodine in the UK, but consumption of plant-based milk alternatives (PBMA) is increasing and these products are often not fortified with iodine. We evaluated the impact that replacing current milk consumption with PBMA would have on iodine intake.

**Methods:**

We used data from the National Diet and Nutrition Survey (2016–2019) for children (1.5–10 years), girls 11–18 years, and women of reproductive age (WRA). We used a dietary modelling approach with scenarios using brand-level iodine-fortification data (0, 13, 22.5, 27.4 and 45 µg/100 mL). Relative to usual diet, we calculated change in iodine intake, and the proportion with intake below the Lower Reference Nutrient Intake (LRNI) or above the upper limit.

**Results:**

For all groups, replacement with PBMA, either unfortified or fortified at the lowest concentration, resulted in a meaningful decrease in iodine intake, and increased the proportion with intake < LRNI; compared to usual diet, iodine intake reduced by 58% in children 1.5–3 years (127 vs. 53 µg/day) and the proportion with intake < LRNI increased in girls (11–18 years; 20% to 48%) and WRA (13% to 33%) if an unfortified PBMA was used. Replacement of milk with PBMA fortified at 27.4 µg/100 mL had the lowest impact.

**Conclusion:**

Replacing milk with commercially available PBMAs has potential to reduce population iodine intake, depending on the fortification level. PBMAs fortified with ≥ 22.5 and < 45 µg iodine/100 mL would be required to minimize the impact on iodine intake. Research is needed on the impact of total dairy replacement.

## Introduction

Iodine is essential for normal thyroid function, thyroid-mediated growth and metabolism at all stages of life [1]. Iodine deficiency, particularly during pregnancy, remains a global public health concern since it increases neonatal mortality and is a preventable cause of cognitive impairment and developmental delays in children [2–4]. Although pregnant women and young children represent vulnerable groups [5], thyroid disorders attributed to mild-to-moderate iodine deficiency are implicated in chronic disease burden in adults [6–8]. In recent years, iodine deficiency has re-emerged as a concern in several European countries, including the UK [9–12]. According to the most recent UK National Diet and Nutrition Survey (NDNS) 2016–2019 [13], the median Urinary Iodine Concentration (UIC) for women (19–49 years) was 97 µg/L, which is below the World Health Organisation’s (WHO) threshold for sufficiency (UIC ≥ 100 µg/L [4]). In general, women have a lower dietary intake of iodine than men and therefore are at greater risk of deficiency [14].

Milk and dairy products are the primary dietary sources of iodine in many European countries, including the UK and Ireland [14]. However, consumers are becoming more aware of the environmental impact of food production, and there are rising concerns over the health effects, and sustainability of current eating patterns [15]. Consequently, plant-based diets are becoming increasingly popular [16], accompanied by the increased popularity and availability of plant-based drinks (e.g., oat, soya, and almond drinks) as an alternative to cow’s milk [17]. According to Mintel market research, 32% of UK adults consumed plant-based milk alternatives in 2021 [18] compared to 19% in 2018 [19], with millennial women (aged 25–40 years) being the main consumers [18]. It is essential to understand the impact that replacing milk with PBMAs may have on iodine intake.

From an iodine perspective, plant-based milk alternatives (PBMAs), unless fortified, are not an adequate replacement for cow’s milk. Studies in the UK, USA and Norway have identified that the iodine content of unfortified PBMAs is low [20–22] and would only provide 2% of the iodine of UK cow’s milk [20]. In 2020, we observed that only 20% of the 146 PBMAs in UK grocery stores were fortified with iodine, of which most were fortified at a lower concentration than cow’s milk [23]. This low iodine content of unfortified PBMAs is significant and it suggests that consumers would be at risk of deficiency unless they incorporated another source of iodine into their diet. A recent study using iodine intake and status data from the NDNS (Years 7–9; 2014–2017) observed that young women were most likely to consume PBMAs (8% of women 16–49 years). Additionally, those consuming PBMAs had a lower iodine intake (94 *vs* 129 µg/day) and status (median UIC: 79 *vs* 132 µg/L) compared to cow’s milk consumers [24]. However, the NDNS years included in this study were when most manufacturers did not fortify their drinks with iodine [20] and therefore there is little information regarding the impact of fortified PBMAs on dietary iodine intake.

The overall aim of this study was to examine the impact on iodine intake and adequacy of transitioning from a diet including milk to one with PBMAs. Specifically, we aimed to evaluate i) the impact of iodine fortification and whether there is an ‘optimal’ level of fortification in PBMAs, and ii) how the choice of PBMA category affects iodine intake and adequacy using current UK retail data. Using data from the UK NDNS (2016–2019, Years 9–11) and brand-level iodine concentration data from PBMAs, several scenarios were modelled, accounting for fortification and the variety of PBMAs available.

## Methods

### Population and food-intake data

The present study used food-intake data from the tenth cycle of the National Diet and Nutrition Survey (NDNS) Rolling Programme (Years 9–11, 2016–2019) and was obtained from the UK Data Archives [13]. The NDNS is a continuous, cross-sectional survey funded by Public Health England and the Food Standards Agency, collecting quantitative information regarding food consumption, nutrient intake assessed via a four-day food diary, and nutritional status of the UK general population (aged 1.5 years and above), with age- and sex-weighting to reflect population distributions. The methodology of the NDNS rolling programme, including the dietary assessment, has been reported in detail elsewhere [25]. Data from subjects who self-reported abstaining from milk consumption (i.e., self-reported as following a vegan diet) or with food-diary data devoid of any milk (i.e. non-milk consumers) were excluded from the present analysis.

The following population groups were included in the current analysis: children 1.5–3 years (n = 306), children 4–10 years (n = 725), adolescent girls 11–18 years (n = 346) and women aged 19–49 years (n = 479). The age groups of 1.5–3 years and 4–10 years were chosen as milk accounts for over half of the iodine intake in children [13], and therefore children rely heavily on milk for iodine intake. Additionally, if parents are consuming PBMAs they may decide to also feed their young children these products. We also included adolescent girls (11–18 years) and women aged 19–49 years as these groups are known to have a lower iodine intake compared to the general population [13, 26]. Some of the reasons for these population groups having a lower iodine intake may be due to avoidance of iodine rich foods such as milk and dairy products [27, 28] and this group is more likely to try a plant-based diet [29, 30]. Additionally, iodine deficiency in utero and early life can cause serious cognitive and motor impairment, making children and women of reproductive age vulnerable subpopulations for iodine deficiency [2].

### Food categorisation to identify milk and milk-containing foods

All food and beverage codes in the NDNS food file that contained milk (e.g. skimmed, semi-skimmed, whole cow’s milk, organic cow’s milk, and goat milk) were identified and labelled as such. Milk codes excluded from the scenarios were: dried milk and follow-on milk, as there is currently no plant-based alternative to these products. Additionally, 1042 recipe codes containing milk were identified, and the percent contribution of milk to each recipe was calculated using the Food Standards Agency (FSA) Standard Recipes Database [31]. For example, for the recipe code “porridge made with semi-skimmed milk”, the proportion of that food code that was milk (80%) was identified and labelled as containing milk for the modelling software.

### Iodine concentration data in milk

The total nutritional contribution of milk to iodine intake was estimated by combining the intake data with the nutritional composition data from UK food tables [32]. Briefly, an aggregate quantity of iodine, per gram, for each milk food or recipe code was multiplied by the intake in grams per day for each participant. Retail milk-iodine concentration can vary due to differences in agricultural practices, such as dairy management system, animal diet, and breed [33], therefore the iodine concentration of milk in the UK food tables ranged from 20–41 µg/100 g.

### Iodine occurrence and concentration data in PBMA used for modelling

The methods used to identify the retail PBMAs on the UK market have been previously reported [23]. Briefly, products were identified through searches on UK supermarket websites in December 2020. Following data collection, the products that were identified as PBMAs were grouped into the following ingredient categories: almond, coconut, oat, pea, rice, soya or other-plant based milk alternatives. They were then complied into a Microsoft Excel database detailing the ingredients listed and the nutritional profile of the PBMAs [23]. For the present study, iodine fortified products were identified in the database by searching the ingredient listings, while iodine concentration was extracted from product labels, information from supermarket and manufacturer’s websites, and from nutritional databases for products.

### Milk replacement scenarios

*Scenario 1: The impact of iodine fortification of plant-based milk alternatives, and the ‘optimal’ level of fortification*

In this scenario, we explored the impact of replacing milk with PBMAs either unfortified or fortified at various iodine concentrations. Five different iodine concentrations were examined: (i) 0 µg/100 mL; (ii) 13 µg/100 mL; (iii) 22.5 µg/100 mL; (iv) 27.4 µg/100 mL; and (v) 45 µg/100 mL (Table 1). These scenarios were based on our 2020 market survey of PBMAs [23]; the iodine concentrations reflected the minimum (13 µg/100 mL), mode (22.5 µg/100 mL), mean (27.4 µg/100 mL), and the maximum (45 µg/100 mL) values of the fortified drinks as per the market survey [23], as well an unfortified value (0 µg/100 mL). By law, organic products are not permitted to use fortificants [34]; therefore, all organic PBMAs are considered as unfortified, i.e., 0 µg/100 mL (Table 1). In this scenario, a conservative approach was followed, assuming that 100% of the population moved to consume PBMAs.

*Scenario 2: The impact of choice of plant-based milk alternative category on iodine intake and adequacy using current retail data*

**Table 1:** Milk-replacement scenarios implemented in the modelling analysis based substituting 100% of the current self-reported milk intake

	Replacement scenario	Probability of being fortified with iodine (%)*	Set or range of iodine concentration used in model (µg/100 mL)**
Scenario 1: Impact of iodine fortification	Unfortified plant-based milk	0	0
	Minimum fortification	100	13
	Mode fortification	100	22.5
	Average fortification	100	27.4
	Maximum fortification	100	45
Scenario 2: Choice of plant-based milk alternative category	All plant-based milk products	20	0–45
	Almond-based products	15	0–45
	Coconut-based products	19	0–30
	Oat-based products	22	0–30
	Other-plant-based products	8	0–25
	Pea-based products	67	0–31
	Rice-based products	0	0–0
	Soya-based products	22	0–45

^*^In both scenarios it was assumed that 100% of the population moved to consume PBMAs. In scenario 1, all PBMAs were fortified with iodine, excluding the unfortified replacement scenario. In Scenario 2, the proportion of products currently on the market that are fortified with iodine is taken into account

^**^In Scenario 1, the PBMAs were fortified at set concentration levels, whereas Scenario 2 reflects the range of fortification concentration currently on the market within each category

In this scenario, we used the 2020 market survey data to examine the probability of a PBMAs being fortified, or not, within any category, while also accounting for the range of iodine concentrations available for purchase (Table 1). As in Scenario 1, 100% substitution was assumed, whereby the total amount of cows’ milk in each participant’s diet was replaced with a PBMA. This scenario was designed to reflect a more realistic situation, accounting for the diversity of products on sale and the range of iodine concentration in fortified PBMAs. For this analysis, the PBMAs were grouped into the following categories: almond, coconut, oat, other-plant, pea, rice or soya-based milk alternatives.

### Data analysis

To characterise current intake of milk and PBMA in the NDNS, intake estimates were generated from individual dietary records detailing food items consumed by each survey participant on each survey day. Values for estimated iodine intake (Mean, SD) represent projected four-day averages for each individual. Multiple days of subject data were used to reflect individual exposure—rather than a single day only, as this may more appropriately represent habitual intake. The approach reflects that taken by the European Food Safety Authority (EFSA) for exposure assessments for the Comprehensive European Food Consumption Database [35]. To control for any selection bias in the associations with nutrient intake, we applied NDNS survey weight factors for individuals for each specific NDNS survey year [13].

Both modelling scenarios were conducted using the web-based software application DaDiet© (Dazult, Version 17.04) [36], which could account for 100% substitution at a range of concentrations as per the market survey (Scenario 1), as well as modelling for the probability of the retail product containing iodine while reflecting the range of concentrations present on the market (Scenario 2).

Iodine intake was estimated from the four-day food diaries; though this method has limitations for the assessment of iodine from iodised salt, this is not of concern in the UK, as there is no iodised salt programme and it is not widely available [37]. The National Cancer Institute method was used to estimate the usual intake of iodine, to assess the percentage of the population meeting the threshold for iodine adequacy [38]. To estimate the prevalence of inadequate iodine intake, we used the UK Lower Reference Nutrient Intake (LRNI) as the UK Department of Health set these cut-offs as the minimum iodine intake required to prevent goitre [39]. The LRNI cut-offs used in this analysis were 40 µg/day for children aged 1–3 years, 50 µg/day for children aged 4–10 years, 65 µg/day for adolescent girls aged 11–18, and 70 µg/day for adults. The risk of excessive iodine intake was evaluated using the tolerable upper intake limit (UL) as a reference value. The UL is defined as the maximum level of total chronic daily nutrient intake unlikely to pose a risk of adverse health effects to humans. The European ULs published by EFSA [40] were used in this analysis, specifically 200 µg/day for children aged 1–3 years, 250 µg/day for children aged 4–10 years, 450 µg/day for adolescent girls aged 11–18, and 600 µg/day for adults. The EFSA UL were selected as the UK does not have an adjusted UL value for children [39].

Given that we know there are differences in modelling scenarios by the research design, typical statistical testing was not appropriate. Thus, to assess meaningful differences in changes in the mean iodine intake for the modelling scenarios, we examined means and their 95th percentile confidence limits. Non-overlapping confidence intervals were deemed meaningful. This approach has been utilised previously in dietary-pattern studies [41, 42].

## Results

### Characteristics of the study sample

The dataset consisted of 1738 participants. Of these individuals, 283 were aged between 1.5 and 3 years, 681 between 4 and 10 years, 295 were females aged between 11 and 18 years and 479 were females aged between 19 and 49 years.

#### Milk as a source of iodine

For all population groups, the key food group contributing to iodine intake was milk, contributing 56% and 41% for children aged 1–3 years old and aged 4–10 years, respectively, and 26% and 20% for adolescent girls and women aged 19–49 years. The average self-reported intake of total milk (all milk) and by milk subgroup (whole, semi-skimmed, skimmed) for milk consumers within the NDNS and by age and gender prior to dietary modelling are shown in Table 2. Overall, in the entire NDNS sample, 89% of the population consumed milk during the survey period with a mean intake of 174 g ± 153 g per day in consumers. Within this, semi-skimmed milk was the largest contributor to total milk intake (60%), followed by whole milk (27%) and skimmed milk (7%). A lower proportion of adolescent girls and women of reproductive age were milk consumers (82% and 84% respectively) than the total population. Males aged 1.5–3 years had the highest mean daily intake of milk (313 ± 205 g/day), while adolescent girls had the lowest (119 ± 104 g/day).

**Table 2:** Daily intake of all milk and by milk subtypes in the NDNS Years 9–11 (2016–2019) for the total population and milk consumers only, stratified by age and gender

Age (years)	All milk			Whole milk			Semi-skimmed milk			Skimmed milk			Plant-based milk alternative		
	% Consumer	Intake (g/day)*		% Consumer	Intake (g/day)*		% Consumer	Intake (g/day)*		% Consumer	Intake (g/day)*		% Consumer	Intake (g/day)*	
		Total population	Consumers only		Total population	Consumers only		Total population	Consumers only		Total population	Consumers only		Total population	Consumers only
1.5–3															
Male	91	285 ± 215	313 ± 205	67	200 ± 217	301 ± 201	47	72 ± 141	153 ± 173	6	2 ± 16	34 ± 59	8	7 ± 62	186 ± 148
Female	93	236 ± 187	253 ± 182	73	186 ± 196	254 ± 188	38	46 ± 93	121 ± 117	8	1 ± 4	9 ± 10	5	5 ± 30	96 ± 102
4–10															
Male	93	198 ± 168	213 ± 166	47	87 ± 154	187 ± 178	65	104 ± 138	161 ± 143	6	5 ± 29	82 ± 84	4	5 ± 36	120 ± 132
Female	94	167 ± 151	178 ± 150	43	61 ± 105	141 ± 119	63	101 ± 139	160 ± 145	9	5 ± 36	52 ± 108	2	2 ± 14	58 ± 62
11–18															
Male	85	178 ± 187	208 ± 186	30	46 ± 107	150 ± 149	62	120 ± 180	196 ± 194	9	8 ± 41	82 ± 109	4	4 ± 28	105 ± 93
Female	82	97 ± 105	119 ± 104	31	29 ± 64	92 ± 86	57	64 ± 95	112 ± 102	8	4 ± 18	46 ± 47	6	9 ± 46	140 ± 120
19–49															
Male	86	138 ± 145	161 ± 144	30	31 ± 98	103 ± 158	65	87 ± 115	135 ± 118	13	11 ± 44	85 ± 89	9	11 ± 52	115 ± 131
Female	84	107 ± 117	128 ± 116	29	29 ± 84	100 ± 133	63	66 ± 95	106 ± 100	14	11 ± 43	77 ± 91	10	11 ± 46	117 ± 98
50–64															
Male	92	179 ± 173	193 ± 172	20	33 ± 137	170 ± 269	76	126 ± 142	165 ± 141	16	19 ± 64	121 ± 252	6	5 ± 25	95 ± 52
Female	94	163 ± 148	173 ± 147	18	18 ± 64	98 ± 120	78	116 ± 146	149 ± 150	26	28 ± 75	108 ± 115	7	6 ± 29	92 ± 66
65 +															
Male	93	201 ± 174	217 ± 171	22	33 ± 111	149 ± 196	73	144 ± 169	197 ± 169	21	22 ± 69	105 ± 116	5	8 ± 42	175 ± 98
Female	93	168 ± 140	181 ± 137	27	27 ± 80	101 ± 126	77	119 ± 130	156 ± 127	20	19 ± 53	99 ± 82	8	10 ± 46	128 ± 106
ALL	**89**	**154 ± 154**	**174 ± 153**	**29**	**39 ± 109**	**132 ± 168**	**67**	**98 ± 132**	**146 ± 137**	**15**	**14 ± 51**	**92 ± 101**	**7**	**9 ± 42**	**118 ± 108**

^*^Figures are mean ± SD

#### Plant-based milk consumption

In total, 7% (n = 204) of the NDNS sample consumed PBMA, however within this sub-group absolute daily consumption was low (118 ± 108 g/day). When examined by age and gender, the highest proportion of consumers was amongst women aged 19–49 years, with 10% reporting consuming a PBMA. Additionally, 8% of male and 5% of female children aged 1.5–3 years consumed PBMAs. As explained in methods, all self-reported PBMA consumption was omitted from the modelling.

### Projected changes in iodine intake

*Scenario 1: What is the impact of iodine fortification on plant-based milk alternatives, and what is the ‘optimal’ level of fortification?*

Table 3 shows the usual intakes of iodine and the potential shifts in daily iodine intake under Scenario 1. The iodine intake on the usual diet (i.e. before modelling) ranged from 97 µg/day in adolescent girls, to 151 µg/day in women of reproductive age. With usual diet, a considerable proportion of girls 11–18 years and women of reproductive age had intake of iodine below the LRNI (20% and 13% respectively).

**Table 3:** The implications of replacing milk with plant-based milk alternatives (with a range of iodine concentrations (Scenario 1)) on usual intake of iodine (µg/day) and the proportion of the population with intakes of iodine below the Lower Reference Nutrient Intake and above the Upper Limit

	Iodine fortification used in model (µg/100 mL)	Children (1.5–3 years) (n = 283)						Children (4–10 years) (n = 681)						Adolescent girls (11–18 years) (n = 295)						Women of reproductive age (19–49 years) (n = 479)					
		Daily intake (µg/day)		< LRNI		> UL		Daily intake (µg/day)		< LRNI		> UL		Daily intake (µg/day)		< LRNI		> UL		Daily intake (µg/day)		< LRNI		> UL	
		Mean	95% CI	%	SD	%	SD	Mean	95% CI	%	SD	%	SD	Mean	95% CI	%	SD	%	SD	Mean	95% CI	%	SD	%	SD
Usual diet		127	120, 134	2	1.4	15	2.6	126	122, 130	4	0.8	5	1.0	97	92, 102	20	3.0	0	0	151	143, 159	13	1.8	1	0.6
Unfortified	0	53*	51, 55	34*	4.0	0*	0	77*	75, 79	28*	2.2	0*	0	72*	69, 75	48*	4.3	0	0	119*	111, 125	33*	2.8	1	0.6
Minimum fortification	13	89*	85, 93	5	2.0	2*	0.7	100*	98, 102	7*	1.1	0*	0.2	87*	83, 91	30*	3.8	0	0	135*	128, 142	20*	2.3	1	0.6
Mode fortification	22.5	116	110, 122	4	1.9	5*	1.4	117*	114, 120	4	0.8	2*	0.6	97	93, 102	23	3.4	0	0	148	140, 156	14	1.9	1	0.6
Average fortification	27.4	129	122, 136	3	1.8	12	2.2	126	123, 129	2*	0.6	3*	0.8	103	98, 108	19	3.1	0	0	154	146, 162	11	1.7	1	0.6
Maximum fortification	45	178*	168, 189	2	1.4	38*	4.0	157*	152, 162	1*	0.3	12*	1.4	123*	116, 130	10*	2.0	0	0	177*	167, 187	7*	1.4	1	0.6

All scenarios replace each food on a gram for gram basis

Lower reference nutrient intake (LRNI): children aged 1–3 years; 40 µg/day, children aged 4–10 years; 50 µg/day, females aged 11–18; 65 µg/day and adults; 70 µg/day. Tolerable upper intake limit (UL): children aged 1–3 years; 200 µg/day, children aged 4–10 years; 250 µg/day, females aged 11–18; 450 µg/day and adults 600 µg/day [35]

^*^Meaningfully different from usual diet due to non-overlapping 95th percentile confidence intervals

For all population groups, replacing milk with PBMAs that were either unfortified or fortified at the minimum iodine concentration (i.e. 13 µg/100 ml) would result in a meaningful decrease in usual iodine intake. The greatest impact was observed for children aged 1.5–3 years where introduction of unfortified milk resulted in a 58% reduction in iodine intake (127 to 53 µg/day).

When replacing milk with PBMAs fortified at 22.5 µg/100 mL, the concentration most frequently present in the 2020 market survey, a 7% decrease in iodine intake was observed in children aged 4–10 years. By contrast, no meaningful difference from baseline was observed for children aged 1.5–3 years, and females aged 11–18 and 19–49 years with use of PBMA at 22.5 µg/100 mL. When replacement was based on PBMA fortified at the average value of 27.4 µg/100 mL, there was no observed meaningful difference in iodine intake in any age group.

When milk was replaced with PBMAs at the maximum fortification level present in the 2020 market survey (45 µg/100 mL), iodine intake would be meaningfully higher than the usual diet in all groups (by 26–51 µg/day). At the maximum fortification level both children aged 1.5–3 and 4–10 years and women aged 19–49 years would be consuming enough iodine to meet the recommended daily intake, however adolescent girls would still have a median iodine intake (at 123 µg/day) below the recommended daily intake.

**Fig. 1:**
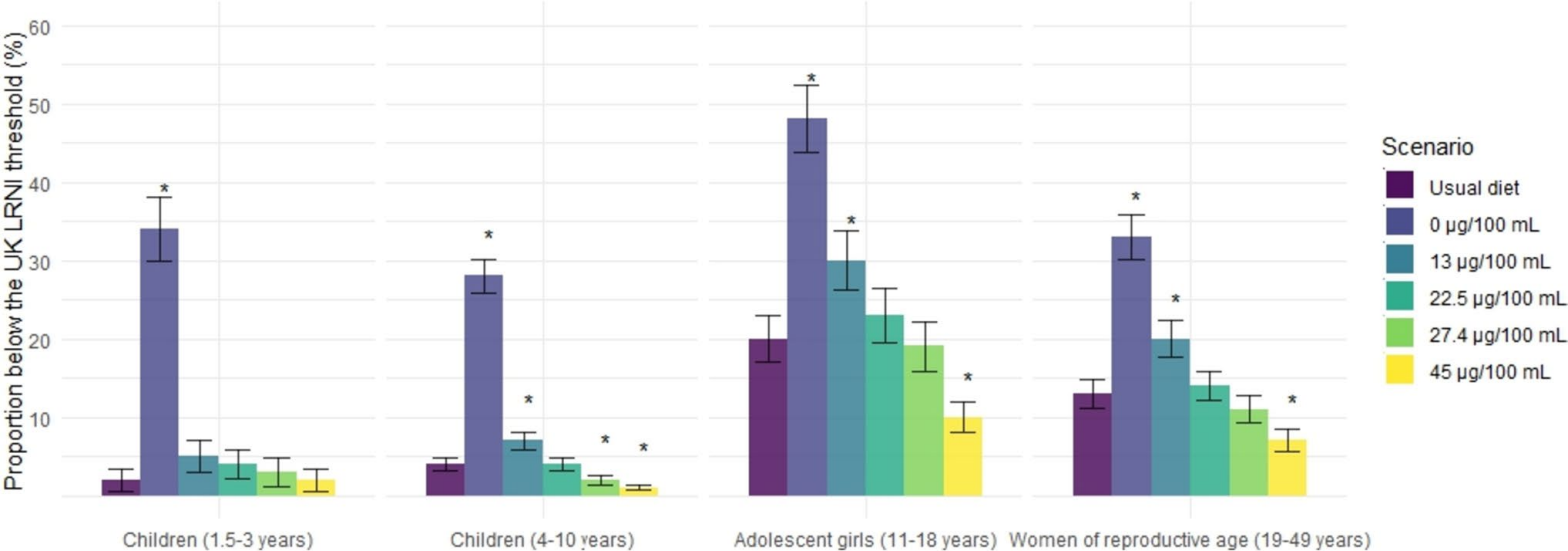
The implications of replacing milk with plant-based milk alternatives based on a range of iodine concentrations (Scenario 1) on the proportion of the population with intakes of iodine below the Lower Reference Nutrient Intake. Lower reference nutrient intake (LRNI): children aged 1-3 years; 40 µg/day, children aged 4-10 years; 50 µg/day, females aged 11-18; 65 µg/day and adults; 70 µg/day. *Meaningfully different from usual diet due to non-overlapping 95th percentile confidence intervals

Across all population groups, replacing milk with an unfortified PBMA would result in a greater proportion of individuals with iodine intake below the LRNI (Fig. 1) than with their usual diet, 28–48% of the population compared to 2–20% with usual diet. Meaningfully different changes in the proportion below the LRNI were also present for children aged 4–10 years, and females aged 11–18 and 19–49 years at the minimum level of fortification.

In terms of the risk of exceeding of the EFSA UL the greatest impact was in children aged 1.5–3 and 4–10 years at the maximum level of fortification (38% and 12% above the UL respectively); replacement with PBMA at 22.5 or 27.4 µg/100 mL had minimal impact on the proportion above the UL. However, 0% of the population exceeded the UK UL (1100 µg/day) at any level of fortification.

*Scenario 2: How does the choice of plant-based milk alternative category impact iodine intake and adequacy using current retail data?*

Table 4 shows the usual intakes of iodine and the potential shifts in daily iodine intake observed with Scenario 2. The modelling shows that any change from usual intake would result in a meaningful decrease in iodine intake across all population groups. Based on the current market, where all rice drinks are unfortified, replacing milk with rice-based drinks would have the greatest impact on iodine intake. However, as 67% of pea-based drinks are fortified, this scenario had the lowest impact on estimated iodine intake. Regardless of which category of PBMA is selected, a change from the usual diet will result in a greater proportion of the population with intake below the LRNI, particularly in adolescent girls and women of reproductive age, and in children aged 1.5–3 and 4–10 years will result in a lesser proportion above the UL.

**Table 4:** The implications of a population shift from milk to different types of plant-based milk alternatives factoring in the availability of iodine-fortified products on the market (Scenario 2) on usual intake of iodine (µg/day) and the proportion of the population with intakes of iodine below the Lower Reference Nutrient Intake and above the Upper Limit

	Products fortified with iodine (%)	Range of iodine concentration (µg/100 mL)	Children (1.5–3 years) (n = 283)						Children (4–10 years) (n = 681)						Adolescent girls (11–18 years) (n = 295)						Women of reproductive age (19–49 years) (n = 479)					
			Daily intake (µg/day)		< LRNI		> UL		Daily intake (µg/day)		< LRNI		> UL		Daily intake (µg/day)		< LRNI		> UL		Daily intake (µg/day)		< LRNI		> UL	
			Mean	95% CI	%	SD	%	SD	Mean	95% CI	%	SD	%	SD	Mean	95% CI	%	SD	%	SD	Mean	95% CI	%	SD	%	SD
Usual diet			127	120, 134	2	1.4	15	2.6	126	122, 130	4	0.8	5	1.0	97	92, 102	20	3.0	0	0	151	143, 159	13	1.8	1	0.6
All milk alternatives	20	0–45	71*	69, 73	15*	2.6	0*	0.3	84*	82, 86	15*	1.6	0*	0.2	80*	77, 83	44*	3.9	0	0	113*	108, 118	27*	2.4	1	0.6
Almond-based	15	0–45	67*	64, 70	18*	3.1	0*	0.4	86*	84, 88	17*	1.7	0*	0.2	80*	77, 83	42*	4.2	0	0	126*	119, 134	29*	2.7	1	0.6
Coconut-based	19	0–30	71*	68, 74	11*	2.6	0*	0	88*	86, 90	18*	1.8	0*	0.2	80*	77, 83	41*	4.2	0	0	126*	119, 133	29*	2.7	1	0.6
Oat-based	22	0–30	66*	63, 69	19*	3.2	0*	0.3	87*	85, 89	18*	1.8	1*	0.3	77*	74, 80	40*	4.2	0	0	126*	118, 134	29*	2.7	1	0.6
Other plant-based	8	0–25	58*	56, 60	22*	3.2	0*	0.3	84*	82, 66	21*	1.9	0*	0.2	77*	74, 80	43*	4.2	0	0	122*	115, 129	31*	2.8	1	0.6
Pea-based	67	0–31	104*	99, 109	6*	2.1	3*	0.9	112*	109, 115	6*	1.1	1*	0.5	85*	81, 89	28*	3.8	0	0	140	132, 148	16	2.1	1	0.6
Rice-based	0	0–0	53*	51, 55	34*	4.0	0*	0	77*	75, 79	28*	2.2	0*	0	72*	69, 75	48*	4.3	0	0	119*	111, 125	33*	2.8	1	0.6
Soya-based	22	0–45	78*	75, 81	12*	2.6	0*	0.3	93*	91, 95	12*	1.4	0*	0.2	80*	76, 84	37*	4.1	0	0	130*	123, 138	24*	2.5	1	0.6

All scenarios replace each food on a gram for gram basis

Lower reference nutrient intake (LRNI): children aged 1–3 years; 40 µg/day, children aged 4–10 years; 50 µg/day, females aged 11–18; 65 µg/day and adults; 70 µg/day. Tolerable upper intake limit (UL): children aged 1–3 years; 200 µg/day, children aged 4–10 years; 250 µg/day, females aged 11–18; 450 µg/day and adults 600 µg/day

^*^Meaningfully different from usual diet due to non-overlapping 95th percentile confidence intervals

## Discussion

In this study, we sought to identify the level of iodine fortification of commercially available plant-based milk alternatives to minimise the risk of iodine deficiency when completely substituting milk. The main finding of our study is that consumer choice will have a substantial impact on iodine intake due to the diversity of PBMAs on sale. Fully replacing milk consumption with an unfortified or organic PBMA would increase the risk of iodine insufficiency across both children and females aged 11–18 and 19–49 years. However, a PBMA fortified to either 22.5 µg/100 mL or 27.4 µg/100 mL would seem to be an adequate replacement for milk in terms of iodine intake, suggesting that the optimal concentration for iodine fortification is approximately 27.4 µg/100 ml. When we modelled iodine intake while considering the probability of consumers selecting an iodine-fortified product based on the current market offering, we identified a meaningful decrease in iodine intake across all age groups.

The results of this present analysis indicate that a transition away from milk and towards PBMAs are likely to affect iodine status in the UK, particularly in those population groups already at risk of deficiency, such as women of reproductive age and adolescent girls (13% and 20 below LRNI with usual diet respectively). Adequate iodine intake is essential to maintain thyroid hormone production, not only to prevent thyroid enlargement (and eventually goiter) but also to ensure thyroidal iodine stores are maximised prior to pregnancy [43]. Iodine deficiency during pregnancy has considerable implications for the developing child and the iodine-intake recommendation is higher for pregnant/lactating women than adults [4]. Emerging evidence suggests that even mild-to-moderate iodine deficiency during pregnancy may be negatively associated with child cognition and behaviour [2]. Whilst pregnant women are not included in our analysis, it is worth noting that none of the fortification scenarios met the WHO recommendation for iodine intake during pregnancy and lactation [4].

While several studies have looked at replacing milk with PBMAs [42, 44–48], very few have investigated the impact on iodine intake. Our results revealed that replacing milk consumption with an unfortified or organic PBMA would increase the risk of iodine insufficiency across all age groups. Overall, the results of our study are in accordance with earlier scenario studies that assessed the effect of PBMAs on iodine intake from the UK [49] and France [50]; The UK study by Clegg et al. [49], also used data from the NDNS (2014–2016) to assess the effects of replacing milk with different categories of PBMA, but only used average consumption data for each age group from the NDNS report, not raw data for individual values from the food diaries as done here. The authors of the earlier study identified 6 iodine-fortified products available in the UK, one coconut-based drink and five legume-based drinks which were used in their models. When replacing the average milk consumption with the coconut-based drink (13 µg/100 mL), they identified significantly lower iodine intake across all age groups. However, when replacing milk with the mean iodine concentration of fortified legume-based milk alternatives (26.3 µg/100 mL) they did not identify a significant change in iodine intake. Similar results were found in France, using data from the French Third Individual and National Study on Food Consumption Survey; Salomé et al. [50] reported that replacing milk with unfortified PBMAs would decrease the probability of achieving an adequate iodine intake [50]. However, this study did not include any iodine-fortified products in their modelling. Our study provides an additional dimension to the previous studies by showing the impact of different levels of fortification, including organic/unfortified products, and takes in to account the probability of selecting an iodine-fortified product making it more reflective of the situation facing consumers.

The results of the current study also demonstrate the importance of considering realistic situations regarding the iodine fortification of PBMAs. In particular, it highlights that the current rate of iodine fortification of PBMAs is not sufficient for total replacement of milk. However, the market for PBMA products is developing rapidly, as highlighted by the differences in iodine-fortified products identified by Clegg et al. in July 2020 and the products identified in December 2020 used in the current analysis [23]. We used data from 29 iodine fortified PBMAs to create our scenarios, which included the most commonly used level of iodine fortification at 22.5 µg/100 mL. Manufacturers often choose this concentration as it is the amount required to be labelled a source of iodine [51] (100 ml provides 15% of the adult RNI); our analysis suggests that this concentration (22.5 µg/100 mL) is an adequate replacement for the iodine in milk, though still resulted in a meaningful difference in iodine intake in children 4–10 years.

The iodine concentration of conventional milk is highly variable as seasonality and farming practices can affect milk iodine concentration; summer milk has been shown to have a lower iodine content than winter milk [33, 52]. As a result, milk iodine concentration can range from 9.5 µg/100 g to 45 µg/100 g, depending on the season [33]. Our study highlights that the nutritional implications of replacing milk with PBMAs are not identical across all at-risk population groups supporting the need for individual variability when making dietary recommendations. While adolescent girls and women of reproductive age may benefit from a higher level of fortification, PBMAs fortified at a concentration of 45 µg/100 mL of iodine may be too high for regular consumption in young children, particularly those under the age of 3 years as the proportion of those with iodine intake over the upper limit increased to 38% (from 15%). While this level of fortification is similar to the iodine concentration of winter milk, consuming a PBMA throughout the year may increase the risk of excess iodine intake in young children. However, the upper limit for children is extrapolated from the adult value and is not based specifically on evidence of direct harm to children. Therefore, the risk of excess iodine intake in this age group is not well known but excess iodine intake can increase the risk of developing iodine-induced thyroid dysfunction [53].

Surveys indicate that although many consumers perceive PBMAs as healthy, they are unaware that these products are nutritionally different to milk, and 23% even consider PBMAs healthier than milk [18]. Our study has highlighted that even if consumers know the nutritional differences, achieving nutritional equivalence is complex, as fortification practices between products vary greatly. In light of the present results, and as the popularity of PBMAs continues to grow, it is important that iodine fortification is mentioned alongside calcium when including PBMAs in public health guidelines, such as the UK’s Eatwell Guide [54] or the British Dietetic Association’s One Blue Dot policy [55]. With the current widespread availability and intake of PBMAs likely to increase, developing recommendations related to its consumption for the population overall and for specific population groups would be a worthwhile inclusion in future dietary guidelines. Countries could also consider other policy strategies, such as using iodised salt in salt-containing industrial food products (such as bread) and recommending iodine supplementation to all pregnant women—strategies not currently in place in the UK.

In the UK, there has been a reduction in milk consumption in recent years [56], especially in population groups such as women of reproductive age [18, 30]. In this study, milk is the primary dietary source of iodine in all four cohorts, although consumption varied between groups. Young children were the most reliant on milk as a source of iodine while adolescent girls and young women were less reliant. This may be in part due to the rise in popularity of PBMAs with adolescent girls and young women [57]. In our sample, baseline consumption of PBMAs was low. However, at baseline, 9% of women aged 19–34 opted for a PBMA. This number is likely to increase over time as consumer interest surrounding sustainable plant-based diets continues to increase.

### Strengths and limitations

The use of raw food diary data from the latest NDNS was one of the strengths of the current modelling analysis due to the quality of the dietary data collected, which underwent rigorous quality checks, including post-collection and post-data entry checks, and the data is weighted to be representative of the UK population. The use of this data allows for the use of these results in a UK setting by policy makers and public health agencies. An important strength of this analysis is the use of statistical modelling to estimate the ‘usual intakes’ of iodine resulting in a better estimate of the true distribution of usual intakes with shorter tails at the upper and lower ends, therefore, improving the estimates of the proportions of the population with intakes above or below a particular reference value (e.g. LRNI or UL) which would otherwise be overestimated [58].

As to weaknesses, there are several uncertainties that may have affected the calculated exposure assessment results (Table 5). Each potential source of uncertainty has been considered qualitatively as recommended by EFSA [35]. Several sources of potential under and over estimation were identified in association with food consumption data and iodine concentration data utilised in the assessment. The present study was a theoretical approach focussing solely on the replacement of milk with PBMAs without considering changes in the consumption of other animal-based foods, including other dairy products such as cheese and yoghurt. Although this study used a large set of PBMAs (146 products) whose full and detailed nutrient composition was available, we may not have fully captured the PBMA market as it is dynamic. Nevertheless, this set of PBMAs was diverse and contained the principal types of substitutes. Consumer preferences and brand loyalty were not considered in this study. Brand loyalty is defined as the tendency of consumers to purchase and consume the same foods repeatedly [35] and can lead to high exposure by brand-loyal consumers if the product contains a high concentration of iodine. This is a source of uncertainty in the current analysis, the direction of the effect is based on level of fortification of these products. Additionally, iodine intake was likely overestimated in the present study, since the possible losses of iodine from preservation and cooking were not considered; we could not apply retention factors for the estimation of iodine in the PBMA after use in cooking as there are no data on these losses in PBMA. Finally, we did not consider plant-based cheese or yoghurt alternatives in the dietary scenarios, and some consumers may switch all their dairy products to plant-based versions, not just milk, leading us to underestimate the potential change to total iodine intake.

**Table 5:** Qualitative evaluation of the influence of uncertainties based on EFSA protocols [35]

Source of uncertainty	Direction and magnitude ^a^
Food consumption data	
Representativeness, Mis-/under-reporting	±
Use of a 4-day food diary to extrapolate chronic intakes	±
Inaccuracies in data on recipe composition	±
Iodine concentration data	
Availability of analytical data in final products (not reported values from manufacturers)	±
Overages on labels	±
Effect of processing, storage or cooking on iodine	±
Brand loyal intake assessment	+ + ± –

^a^ +  + , +  + , +  +  + are the uncertainty likely to cause small, medium or large overestimates of exposure

–, – –, – – – are the uncertainty likely to cause small, medium or large underestimates of exposure

Future studies should take the aspect of bioavailability into consideration, especially in studies concerning population groups who are vulnerable to iodine deficiency, including premenopausal women, pregnant or lactating women, strict vegetarians/vegans, young children, or those with a milk allergy. Even when fortified to similar levels to conventional milk, PBMAs may not be equivalent because the type of ingredient used to fortify the PBMA could affect bioavailability. Both potassium iodide and seaweed have been used as iodine sources in PBMAs [20] yet the latter has been found to be less bioavailable [59]. In this study we have assumed a 100% switch of milk with PBMAs, however, only around half (47%) of the PBMA consumers in the NDNS data set consumed alternatives 100% of the time. Of the consumers who consumed both milk and PBMAs, they were more likely to consume PBMAs in the home, and often consumed the milk in the workplace or at a friend’s house where PBMAs might not be available. Therefore, future studies should also consider the impact of a partial replacement of milk with PBMAs to account for this type of consumer. Our study focused on iodine, but cow’s milk is an important source of other micronutrients, including vitamins B2 and B12, and as our market survey [23] has highlighted that PBMAs are not always fortified with these either, further modelling studies could consider other nutrients.

## Conclusion

Our modelling results suggest that the replacement of milk with commercially available PBMAs has great potential to affect intake of iodine intakes at a population level, depending on the level of fortification. We identified that fortification at approximately 22.5–27.4 µg/100 mL (and < 45 µg/100 mL) would be sufficient to minimize the impact of transitioning from milk to PBMA on iodine intake. However, the majority of PBMAs are still not fortified with iodine, and the likelihood of consumers selecting an unfortified product is high. Individuals who choose to consume unfortified or organic PBMAs in place of milk will need to be more mindful of their iodine intake. If these PBMAs are consumed as part of a diet with other iodine-rich foods such as fish and eggs, there may be less risk of deficiency, but those following a plant-based diet such as a vegan diet would be at a higher risk of deficiency when consuming unfortified PBMAs. Consequently, if PBMAs are to be consumed in place of milk, public health messages need to ensure that alternative sources of iodine are clearly signposted.

## Data Availability

The data underlying the results presented in the study are available from the UK Data Service: (https://beta.ukdataservice.ac.uk/datacatalogue/studies/study?id=8159), (https://beta.ukdataservice.ac.uk/datacatalogue/studies/study?id=6533).
